# Revolutionary discoveries and the disruption index: Probing the relationship

**DOI:** 10.1371/journal.pone.0360037

**Published:** 2026-10-27

**Authors:** Lutz Bornmann, Robin Haunschild, Søren Riis Paludan, K. Brad Wray

**Affiliations:** 1 Science Policy and Strategy Department, Administrative Headquarters of the Max Planck Society, Munich, Germany; 2 Max Planck Institute for Solid State Research, Stuttgart, Germany; 3 Department of Biomedicine, Center for Immunology of Viral Infections, Aarhus University, Aarhus C, Denmark; 4 Department of Mathematics, Centre for Science Studies, Aarhus University, Aarhus C, Denmark; National Research Council: Consiglio Nazionale delle Ricerche, ITALY

## Abstract

A few years ago, the Disruption Index (DI) was proposed as a tool for identifying disruptive research. However, it remains unclear whether the index actually measures what it claims to measure. Our proposed survey plans to examine the extent to which the papers identified by the DI as especially disruptive are the same as the papers that scientists working in a field identify as especially disruptive. We, thus, aim to test an assumption about what the DI measures, specifically, that papers with high DI values are papers reporting revolutionary discoveries. We will focus on the field of immunology. Our survey data will be drawn from researchers who have published many papers in immunology.

## Introduction

There has been intense interest in the Disruption Index (DI) since the publication of Wu, Wang and Evans [1] [see, for example, 2]. Wu, Wang and Evans [1] report that smaller researcher teams are more likely to produce more disruptive research papers (papers with larger DI values) than larger researcher teams, who tend to produce less disruptive research papers (papers with lower DI values). Based on publication and citation meta data, the DI measures the degree to which a scientific paper departs from the existing scholarly literature and thus breaks new ground. The DI is frequently contextualized within Thomas Kuhn’s theory of paradigm shifts [3], which characterizes scientific progress as a sequence of revolutionary transitions rather than a linear accumulation of facts. Insofar as the DI measures discontinuity in the scientific literature in a field, it may be an effective means for identifying revolutionary scientific breakthroughs which are also conceived as discontinuous developments. Revolutionary scientific discoveries are the sorts of discoveries that are field-changing. Disruptive innovation is identified as research that introduces “dramatic changes with an overthrow of established thinking” [4, p. 4731], thus aligning with Kuhn’s description of revolutionary science. This stands in contrast to ‘normal science,’ which is grounded in a paradigm-bound tradition and consensus-oriented convergent thinking.

As gathering the publication and citation data needed for determining the DI of a research paper is a complex process, and requires large sets of bibliometric data, the DI is aptly described as an analyst’s concept, rather than an actor’s concept. Scientists working in a specific field, the actors, are unlikely to know the DI of any given paper. In fact, they may not be able to effectively guess the DI of papers in their field. Only scientometricians and others who work with such data on a regular basis, the analysts, are apt to be familiar with the DI and to fully comprehend what exactly it is measuring. Surveys or interviews can be used to get at the actors’ understanding of the dynamics of their research field, and specifically their sense of which research papers constitute revolutionary contributions to their field. One impediment with conducting such a survey is that different scientists may have different understandings of what constitutes a revolutionary contribution. Indeed, it is widely recognized that the concept ‘scientific revolution’ is a contested term [5]. But this impediment is not insurmountable. In principle, scientists could be given a definition or illustration of a typical or paradigmatic scientific revolution as a means for focusing their attention on the salient features of a revolutionary change in science before their opinions are solicited.

Our aim in the planned study is to investigate whether the DI is an effective measure of revolutionary discoveries. While this was always assumed in previous studies using the DI, it was inadequately empirically verified. We will do this by comparing bibliometric data to survey data in which scientists in a specific field identify which publications have had a revolutionary impact. Our aims are to deepen our understanding of the extent to which the DI measures the same thing that Kuhn [3] regards as revolutionary.

Our study will focus on the field of immunology. One of the co-authors of this preregistration is an active researcher in immunology and can therefore provide a professional assessment of the research in this field. To assist non-expert readers in conceptualizing what constitutes a ‘radical’ breakthrough in this specific medical domain, the final manuscript reporting the results of this preregistered study will include a brief historical overview of the field. Drawing on the expertise of our co-author, we will describe established historical examples of revolutions in immunology. This contextualization will illustrate the theoretical expectations of what a well-functioning DI should ideally capture, without priming the survey respondents. We have not provided an example of a revolution in immunology in the pre-registration because we do not want to influence the answers we get from the immunologists in the survey (they may read the pre-registration).

## Revolutions in science

The theory of scientific development proposed by Kuhn [3] presents the history of science as a non-cumulative process characterized by long periods of stability punctuated by radical transformations. Central to this theory is the concept of a paradigm, which Kuhn later defined as a ‘disciplinary matrix’ encompassing shared symbolic generalizations, metaphysical commitments, and validation standards [6,7]. A paradigm serves as the fundamental foundation for a scientific community, defining both the legitimate problems for a study and the acceptable methods for their solution [3,8]. By providing a shared conceptual framework, a paradigm allows researchers to focus on esoteric puzzles with great efficiency, effectively ‘insulating’ the community from broader societal problems that do not fit the established puzzle form [9,10].

Science conducted within the constraints of an accepted paradigm is defined as ‘normal science’ [3]. During these periods, scientific activity is primarily characterized by ‘puzzle-solving,’ where researchers aim to articulate and extend the existing paradigm rather than to produce fundamental novelties. This phase exhibits a high level of consensus, as most members of a field accept the truth and legitimacy of the reigning paradigm [11]. Consequently, knowledge during periods of normal science grows in a steady, cumulative fashion, where recent literature builds directly upon earlier foundational work [6,12]. This cycle of stability is inevitably interrupted when researchers encounter ‘anomalies,’ which are empirical or theoretical findings that cannot be resolved within the confines of the established paradigm [3]. While isolated anomalies are often brushed aside, an accumulation of such contradictions precipitates a state of ‘crisis.’ During a crisis, the consensus on basic concepts and methods in a scientific community breaks down, and researchers begin to challenge the fundamentals of their field [8]. Sometimes a scientific community can normalize an anomaly with the conceptual resources of the long-accepted paradigm. But such crises are also resolved by a ‘scientific revolution,’ a “non-cumulative developmental [episode] in which an older paradigm is replaced in whole or in part by an incompatible new one” [13, p. 92].

Revolutionary changes involve what Kuhn [14] describes as ‘taxonomic changes,’ reordering scientific concepts so that the relationships present in the old theory are no longer preserved in the new theory [12]. This leads to ‘incommensurability,’ where competing paradigms represent incommensurable ways of science that cannot be meaningfully compared through a neutral, paradigm-independent standard [15,16]. Because proponents of different paradigms essentially “live in different worlds” [17, p. 121], the choice between them is often driven by a combination of logical, aesthetic, and social factors rather than a simple appeal to the truth [18]. Kuhn [3] ultimately argued that scientific progress does not move toward a fixed goal of objective truth but is a process similar to biological evolution, pushed from behind by the successes of its problem-solving capabilities [19].

## Research on and with the disruption index

Leibel and Bornmann [2] provide a survey of the vast literature on the DI. The DI, also known as the CD index (Consolidation-disruption index), is a network-based metric designed to measure the degree to which a scientific work destabilizes or consolidates its research field [20]. A disruptive work is defined as one that “introduces something new that eclipses attention to previous work upon which it has built” [1, p. 378], effectively rendering earlier foundational ideas less relevant to future researchers [21]. Conversely, a consolidating work reinforces existing discoveries, causing subsequent publications to cite the focal work alongside the earlier research it built upon [22].

The DI typically ranges from −1 for most consolidating, and thus conservative, to 1 for most disruptive, and thus innovative [20]. The DI measures the extent to which a focal paper makes its own references ‘obsolete.’ Disruptive papers are characterized by future research that cites the focal paper but ignores its predecessors, suggesting the new work has opened a new trajectory. Consolidating papers are characterized by future research that cites the focal paper alongside its predecessors, suggesting the work builds upon and reinforces existing ideas. The DI is calculated based on the citation patterns of a focal paper and its set of references:


$$\begin{array}{c} DI=\frac{N_{F}-N_{B}}{N_{F}+N_{B}+N_{R}} \end{array}$$

where *N*_*F*_ is the number of citing papers that cite the focal paper but none of its references, *N*_*B*_ is the number of citing papers that cite both the focal paper and at least one of its references, and *N*_*R*_ is the number of citing papers that cite at least one of the focal paper’s references but not the focal paper itself.

The DI has been applied in various specialized contexts, including urology, radiology, and pharmacy, to identify disruptive publications that traditional citation counts might overlook [23–25]. In trauma and pediatric surgery, for instance, the DI has been found to capture paradigm-shifting studies that show little to no correlation with citation counts [26,27]. Broader bibliometric analyses across decades reveal a secular decline in the average disruptiveness of scientific and technological knowledge [20]. In the field of business and entrepreneurship, average disruption scores have reportedly declined significantly since the 1960s [28]. This trend is often linked in these studies to the ‘burden of knowledge,’ where researchers must accumulate increasing amounts of information to reach the scientific frontier [29]. There are also other explanations, which are summarized by Bornmann and Funk [30].

The primary advantage of the DI is its ability to reveal the nature of scientific influence by examining how future research makes use of the research cited by a focal paper [1]. However, the index faces significant challenges regarding its validity and robustness. Studies on the validity of the DI have produced mixed results. For example, while some find that Nobel Prize-winning papers are highly disruptive, other evidence suggests these landmark works are not necessarily more disruptive than median benchmark articles [31,32]. Furthermore, the index is highly sensitive to the length of the citation window and the number of references in a focal paper [33]. The decline in disruption scores over time that has been found in several studies may also be partially attributed to ‘citation inflation’ and increasingly long reference lists, which create a systematic temporal bias [34]. To mitigate these disadvantages, several variants of the DI have been proposed. Leibel and Bornmann [2] provide an overview of the variants. Currently, there is no consensus that any of the variants is the superior measure.

As an alternative to the standard DI, Bornmann, Devarakonda [35] introduced the DI5. This variant restricts B-type citing papers (*N*_*B*_) to those that reference at least five of the focal paper’s cited references. Consequently, the DI5 emphasizes citing works that build more substantially upon the focal paper’s foundational research, filtering out papers that only cite a few highly cited references. Thus, compared to the standard DI, the DI5 is less susceptible to the referencing habits of the focal paper’s authors and instead better reflects the actual citation behaviour of subsequent research.

The DI_5_ is defined as


$$\begin{array}{c} {DI}_{\text{5}}=\frac{N_{F}-N_{B}^{\text{5}}}{N_{F}+N_{B}^{\text{5}}+N_{R}} \end{array}$$

For the present study, we employ both the DI and the DI5. We selected the DI as it is the original and most widely adopted indicator in the literature. The DI5 was chosen because it has demonstrated a strong performance among all variants in prior validation studies.

## Data and methods

There are two sets of data that will be collected and compared in this study.

First, we will conduct a bibliometric analysis of the literature in immunology to determine which papers in the field have the highest DI. We have already conducted and published a preliminary study of this literature [36]. One of the concerns we had with our previous study is that methodological papers which were not specific contributions to immunology tended to get very large impact. For example, the paper by Lowry, Rosebrough [37] has been cited more than 355,000 times (in many fields). In fact, this is the most cited paper in history [38,39]. In this current study, we will set aside methodological papers that, though important in immunology, are equally or more important in other scientific fields. Thus, we aim to determine which papers in immunology specifically have the highest DI.

We will use a five-year citation and referencing window in the DI calculation following recommendations by Chen, Bornmann and Bu [33]. Following the recommendations of Leibel and Bornmann [2], we will require a minimum number of citations and linked references for the set of focal papers. DI values are already pre-calculated for our Web of Science (WoS, Clarivate) snapshot from April 2025. Our local WoS snapshot has been derived from the Science Citation Index Expanded (SCI-E), Social Sciences Citation Index (SSCI), Arts and Humanities Citation Index (AHCI), Conference Proceedings Citation Index – Science (CPCI-S), and Conference Proceedings Citation Index – Social Science & Humanities (CPCI-SSH) provided by Clarivate via the German “Kompetenznetzwerk Bibliometrie” (Competence Network Bibliometrics, see also https://bibliometrie.info/en/about-kb/) funded by Federal Ministry for Education and Research (BMBF) (grant 16WIK2101A).

To account for the recognized mathematical limitations of the standard DI – such as its sensitivity to the length of a paper’s reference list – we will implement robustness checks. Acknowledging the risk that the DI might overestimate the disruptiveness of papers that cite few literature, our statistical models will incorporate the focal paper’s reference count and citation count as control variables. We will additionally consider the DI5 indicator. By comparing both the DI and the DI5 variant with our survey results, we aim to identify whether this variant is more robust against DI flaws while successfully capturing the actor’s concept of a scientific revolution.

Second, we will collect survey data from active researchers in immunology. Our subjects will be a subset of the immunologists drawn from the most frequently publishing scientists in the field. Screenshots of the planned survey are shown in Figure A1, Figure A2, Figure A3, Figure A4, Figure A5, and Figure A6 in the S1 Appendix. We will use Scopus (Elsevier) for determining the most frequently publishing scientists due to its high quality of author name disambiguation. Our Scopus snapshot from April 2025 was also provided by the German “Kompetenznetzwerk Bibliometrie” (Competence Network Bibliometrics, see also https://bibliometrie.info/en/about-kb/) and funded by BMBF (grant 16WIK2101A). We found 18,256 authors with at least 30 publications between 2005 and 2024 in the following fields: ‘General Immunology and Microbiology,’ ‘Immunology and Microbiology (miscellaneous),’ ‘Immunology,’ and ‘Immunology and Allergy.’ We can expect that these authors, having published at least 30 articles in these fields, are highly knowledgeable about the field of immunology. Depending on the actual return rate, we will lower the 30 articles threshold of number of papers per author or include related disciplines to contact additional immunologists for our survey.

The Scopus database has an email address for 18,139 of the 18,256 authors. We will use these email addresses in a first round of a survey to determine the likely return rate. Currently, we expect a return rate of completed surveys of around 2,8% based on previous survey experiences [40]. Given this expected response rate, we will systematically assess the representativeness of our sample. We will query the Scopus metadata to compare the general academic characteristics (such as citation and publication counts) of our respondents against the overall target population to test for non-response bias. Furthermore, to capture diverse perspectives and ensure the validity of our findings across different strata of the community, we will segment the target population into distinct academic performance groups (e.g., classifying authors based on the percentage of highly cited – top 10% - publications). To ensure the strict anonymity promised to our participants, these segments will be appended to the survey data as broad categorical variables before all personal identifiers are permanently deleted. In our subsequent statistical evaluation, we will examine the mean and standard deviation of the survey responses across these subgroups to determine if the perception of revolutionary discoveries varies systematically by academic impact level.

To ensure that our subjects share the same conception of a revolutionary discovery, we have constructed a brief narrative describing a revolution in early-20^th^ Century chemistry and identified a sample of papers that are associated with this discovery (Figure A3 in S1 Appendix). The narrative is based on earlier work by Wray [16]. It concerns the discovery of isotopes and atomic numbers, which led to the current conception of chemical elements as defined essentially by their atomic number. The subjects will read the narrative before they answer the survey questions (Figure A4 and Figure A5 in S1 Appendix). The survey asks eight questions. The first question asks the subjects to identify a revolutionary discovery in immunology and to cite a specific paper or set of papers associated with the revolutionary discovery (Figure A4 in S1 Appendix). The remaining seven questions solicit answers on a ten-point Likert scale (Figure A5 in S1 Appendix). The Likert scale will aid us in determining what features of the paper are responsible for its being regarded as revolutionary.

We will not collect any demographic information from our subjects. The subjects of our study will not be manipulated in any way, nor will we divide them into an experimental group and a control group. The survey data will be fully anonymized. Since the Aarhus University Research Ethics Committee confirms that Danish law does not require ethical approval for projects that fall outside the scope of the Danish Act on Research Ethics Review of Health Research Projects and Health Data Research Projects, we choose not to have the pre-registration reviewed by the ethics review board.

## Analytical framework

To align the qualitative and quantitative survey responses with the bibliometric data, we will employ a three-step analytical framework.

First, we will manually extract the specific publications nominated by the respondents in the open-ended free-text field (Figure A4 in S1 Appendix). We will then match these identified papers with our WoS snapshot to retrieve their pre-calculated DI scores.

Second, we will analyze the seven quantitative items rated on the 10-point Likert scale (Figure A5 in S1 Appendix). By examining the descriptive statistics, specifically focusing on the mean and standard deviation, we will validate the degree to which the nominated papers are perceived by experts to embody the theoretical dimensions of a scientific revolution (e.g., the resolution of a crisis, taxonomic changes, or the introduction of new methodologies).

Third, we will align the survey metrics with the DI scores through statistical analysis. We will conduct hypothesis testing to determine whether the papers nominated as revolutionary by field experts exhibit significantly higher DI scores compared to expected baseline distributions in the immunology literature. Furthermore, we will explore correlations between the individual Likert ratings and the DI scores to investigate whether specific perceived dimensions of a revolution align more strongly with DI scores.

## Discussion

The primary objective of the planned study is to evaluate the empirical validity of the DI as a metric for identifying revolutionary scientific discoveries. While the DI has garnered intense interest and is frequently contextualized within the theory of paradigm shifts [3], it remains unclear whether high DI scores truly reflect field-changing, disruptive innovations. By comparing bibliometric data with survey data from active researchers in immunology, this study aims to determine if the ‘analyst’s concept’ of disruption mathematically aligns with the ‘actor’s concept’ of a scientific revolution.

As outlined in the theoretical framework, Kuhn [3] posits that scientific development consists of long periods of normal, cumulative science that are periodically interrupted by revolutionary transitions. During normal science, researchers engage in consensus-oriented puzzle-solving within a shared disciplinary matrix, whereas revolutionary science involves the overthrow of established thinking due to accumulated anomalies and crises. Revolutionary discoveries introduce taxonomic changes and incommensurability, forcing a fundamental conceptual reorganization within the field. The DI attempts to mathematically operationalize this phenomenon by measuring citation dynamics; specifically, it assesses whether subsequent literature cites a focal paper while ignoring its foundational references – signaling a departure from the previous paradigm – or if it cites the focal paper alongside its predecessors, signaling normal, consolidating science.

However, previous research on the DI has yielded mixed results regarding its validity. For instance, while some Nobel Prize-winning papers show high disruptiveness, other landmark works do not distinguish themselves from median benchmark articles. Furthermore, the DI is known to be highly sensitive to parameters such as citation window lengths and reference counts. The widely reported decline in disruption scores over time may be partially driven by systematic biases like citation inflation rather than an actual decrease in revolutionary science. Our study directly addresses these ongoing ambiguities by empirically testing the fundamental assumption that the DI accurately measures revolutions in the sense of Kuhn [3].

Methodologically, this study bridges large-scale bibliometric data from the WoS with qualitative insights drawn from a highly specialized population of immunologists identified via Scopus. An important enhancement in our research design is the exclusion of methodological papers. Historically, methodological works – such as the immensely cited paper by Lowry, Rosebrough [37] – can accrue high DI scores despite lacking domain-specific revolutionary impact in immunology. By filtering out these broad methodological contributions, the study ensures that the calculated DI scores reflect conceptual breakthroughs specific to the field of immunology.

To counteract the inherently contested nature of the term ‘scientific revolution,’ participants are provided with a standardized historical narrative regarding the discovery of isotopes and atomic numbers in early-20^th^ Century chemistry. This case study serves as a conceptual primer, focusing the subjects’ attention on the salient features of paradigm shifts – such as the resolution of anomalies and taxonomic changes – before they are asked to nominate revolutionary papers in their own field. The subsequent evaluation of these nominated papers across a seven-item, ten-point Likert scale systematically breaks down the abstract concept of a revolution into specific theoretical components. By asking experts to rate indicators such as widespread disappointment with previous paradigms, the superiority of new methods, and the emergence of incompatible concepts, the survey captures the multidimensional nature of revolutionary shifts.

The outcomes of this research will yield theoretical and practical implications depending on the degree of alignment observed. If the survey results demonstrate a strong positive correlation with the bibliometric data – meaning that the immunological papers identified as revolutionary by field experts consistently exhibit high DI values – this would provide robust empirical validation for the DI. It would substantiate the claim that the DI effectively captures the obsolescence of prior paradigms and the establishment of new scientific trajectories. Conversely, a divergence between expert consensus and high DI scores would highlight critical limitations in the metric. Such a finding might suggest that actors within a scientific field perceive revolutions differently than citation networks imply. For example, scientists might continue to cite displaced paradigms for comparative or rhetorical purposes, thereby artificially suppressing a truly revolutionary paper’s DI score. A lack of correlation would compel the scientometric community to reassess whether the DI is an accurate proxy for conceptual breakthroughs or merely a reflection of shifting citation behaviors and reference list inflation. Understanding what the DI measures is crucial not only for scientometrics but also for broader societal applications, as the metric may be used by policymakers to allocate public funds and by enterprises to strategically organize research and development [41]. Because evaluations based on the DI (using patent data) can inform decisions regarding public policy and industry restructuring, it is essential to ask whether the index reliably captures true innovation [42]. An uncritical application of the DI can lead to misleading conclusions and ineffective strategies for organizing future science and technology.

As with any empirical investigation, several potential limitations must be anticipated and acknowledged. First, the reliance on an expected survey return rate of approximately 2.8% may introduce response bias. Although the sample is drawn from a highly knowledgeable pool of over 18,000 authors who have published at least 30 articles in immunology-related fields, the respondents might not perfectly represent the broader scientific community. If the return rate falls below our expectations, the planned adjustment of inclusion thresholds or the expansion into related disciplines will be necessary to ensure adequate statistical power. Second, while the chemistry case study is designed to harmonize the participants’ understanding of a scientific revolution, subjective differences in interpretation remain an inherent challenge when dealing with human subjects. Standardizing a complex philosophical definition across thousands of independent actors is inherently difficult. Finally, restricting the analysis to the field of immunology allows for targeted professional assessment by a co-author of this preregistration, but it inherently limits the immediate generalizability of the findings to other scientific disciplines, which may possess different publication cultures and citation dynamics.

In conclusion, this preregistered study will provide a much-needed critical examination of the DI. By bridging the gap between the analyst’s bibliometric calculations and the actor’s lived experience of scientific progress, the findings will deepen our understanding of how revolutionary science can be accurately identified and measured. As science policy increasingly relies on quantitative metrics to capture innovation and guide research funding, ensuring that these metrics genuinely reflect structural scientific breakthroughs is of paramount importance. Ultimately, this research will either solidify the DI’s standing as a valid indicator of scientific revolutions or highlight the necessity for new, more context-sensitive bibliometric approaches.

### Declaration of generative AI in scientific writing

During the preparation of this work the authors used Gemini Pro 3.1 to improve language and readability. After using this tool, the authors reviewed and edited the content as needed and take full responsibility for the content of the publication.

## Supporting information

S1 Appendix Figure A1: Cover letter. Figure A2: Entry page of the survey. Figure A3: Second page of the survey with a case study as an example for a revolution in chemistry. Figure A4: Third page of the survey with a free-text field to describe a revolution that occurred in immunology. Figure A5: Ten-point Likert scale to rate agreement or disagreement regarding seven statements. Figure A6: Final page of the survey that confirms that the responses have been recorded.
(ZIP)
